# Solicitude: balancing compassion and empowerment in a relational ethics of hope—an empirical-ethical study in palliative care

**DOI:** 10.1007/s11019-015-9642-9

**Published:** 2015-05-06

**Authors:** Erik Olsman, Dick Willems, Carlo Leget

**Affiliations:** Section of Medical Ethics, Department of General Practice, Academic Medical Center, University of Amsterdam, P.O. Box 22700, 1100 DE Amsterdam, The Netherlands; Department of Ethics of Care, University of Humanistic Studies, Utrecht, The Netherlands

**Keywords:** Hope, Ethics, Palliative care, Qualitative research, Solicitude, Empowerment, Compassion

## Abstract

The ethics of hope has often been understood as a conflict between duties: do not lie versus do not destroy hope. However, such a way of framing the ethics of hope may easily place healthcare professionals at the side of realism and patients at the side of (false) hope. That leaves unexamined relational dimensions of hope. The objective of this study was to describe a relational ethics of hope based on the perspectives of palliative care patients, their family members and their healthcare professionals. A qualitative longitudinal method based on narrative theories was used. Semi-structured interviews on hope were conducted with twenty-nine palliative care patients, nineteen friends or family members, and fifty-two healthcare professionals, which were recorded and transcribed. Data on hope were thematically analyzed. The researchers wrote memos and did member checking with participants. When participants spoke about hope, they referred to power and empowerment, like the powerful bonding of hope between patients and physicians. They also associated hope with the loss of hope and suffering. Several participating healthcare professionals tried to balance both sides, which involved acknowledgment of hope and suffering. Hope and power were reflected in the ethical concept of empowerment, whereas suffering and the loss of hope were reflected in the ethical concept of compassion. Empowerment and compassion can be balanced in solicitude. In conclusion, a relational ethics of hope requires solicitude, in which healthcare professionals are able to weigh empowerment and compassion within particular relationships.

**Electronic supplementary material:**

The online version of this article (doi:10.1007/s11019-015-9642-9) contains supplementary material, which is available to authorized users.

## Introduction

The ethics of hope has often been described within the context of truth telling, for instance when healthcare professionals have to break bad news. They may have to tell patients that a treatment failed to work or that patients suffer from a serious disease. In situations like these, healthcare professionals may want to tell the truth and respect patient autonomy, while simultaneously maintaining patients’ hope (Olver 2005; Ruddick 1999). This perspective may be connected to deontological theories, in which healthcare professionals experience a conflict of duties.

However, “hope versus truth” only reflects a realistic perspective on hope (AUTHOR), which may easily place healthcare professionals at the side of realism and patients at the side of (false) hope. That leaves unexamined relational processes underlying shared hope (Buiting et al. 2011; The et al. 2000). Furthermore, only a few authors have addressed relational-ethical dimensions of hope (Knibbe and Verkerk 2007, 162–181; Simpson 2004; Urban Walker 2006, 40–71), and these dimensions have hardly been examined empirically.

The objective of our study was to describe a relational ethics of hope based on the perspectives of palliative care patients, their family members and their healthcare professionals. The central question was as follows: what does a relational ethics of hope consist of? The research question was answered from an empirical-ethical point of view (Adler and Shaul 2012; AUTHOR), which involved that we examined what participants stated about hope and how hope played a role within their relationships. In the discussion section, the empirical findings will be connected to the work of Nussbaum, Erikson and Ricoeur, in order to articulate the ethical dimensions of the empirical findings, leading to a relational ethics of hope.

The findings of this study may help healthcare professionals to see various sides of what is at stake when they work with hope, which may widen their reflective equilibrium (Urban Walker 2009), which supports them to sensitively and flexibly attune to the needs of patients. Furthermore, the results may support healthcare professionals to deal with hope in such a way that the quality of their relationship with patients is maintained or reinforced.

### Method

Being part of a longitudinal qualitative study on hope in palliative care (Olsman et al. 2014), this paper focuses on the ethics of hope.

## Theoretical underpinnings

Our approach was informed by narrative theories, in which relational dimensions, among other dimensions, are significant (Charon and Montello 2002; Frank 2013; Nelson 1997). Narrative theories include phenomenological approaches, in which the lived experiences of participants are paramount.

### Data collection

Semi-structured interviews on hope were conducted with palliative care patients, their family members and healthcare professionals. Sampling of participants aimed to obtain variation in gender and age. Eligible participants were 18 years of age or older and they were informed about the study both orally and by letter.

Nurses, chaplains and physicians were approached through newsletters and palliative care networks. During the interview they were asked to tell about their own hope and the hope of their palliative care patients and patients’ family members. Then, participating healthcare professionals were informed about the sampling of patients and they were asked to approach eligible patient participants.

When patients agreed to be approached by the researchers, they received information about the study. They were asked for their preference for being interviewed alone or with a family member or friend. In the latter case, the friend/family member was also informed about the study. During one interview the interviewer had the idea that the patient did not feel free enough to express herself because of the presence of her partner. In this case, the interviewer proposed to do the next interview apart from each other. Most of the couples that were interviewed together, however, were able to express differences in views between themselves and their partner. After the interview patient participants were asked for written and oral consent for interviewing their healthcare professional and/or their family members about them.

Palliative care patients were included when they suffered from incurable cancer, severe chronic obstructive pulmonary disease (COPD), or from severe heart failure (HF). Severe COPD means gold (Global Initiative for Chronic Obstructive Lung Disease) 3 or 4, and severe HF means NYHA (New York Heart Association) III or IV. These three diseases were selected because they are some of the most prevalent causes of death in Western countries and because they follow different trajectories (Murray et al. 2005).

Interview guides were developed on the basis of three pilot interviews and on the basis of literature on hope in healthcare (see Supplementary File 1). The interviews were audiotaped and transcribed. There are various definitions and approaches of hope in healthcare and philosophy (Eliott 2005; Herrestad et al. 2014; Kylmä et al. 2009; Olver 2012; Paley 2014; Stempsey 2015). However, we were mainly interested in participants’ ideas about hope and wanted to approach their interpretations of hope without being driven by theory on hope on forehand.

Patient participants were interviewed three times with average intervals of 6 months. The main reasons for drop out were fatigue and death. Patient participants agreed to be called every 6 weeks and during these conversations the question was asked whether their hope had changed. If their hope had changed, a subsequent interview was planned earlier than the scheduled 6 months, in order to stay close to participants’ experiences and understandings of change (Neale and Flowerdew 2003). Interviews with hospice patients were conducted every month because of their limited prognosis. We tried to interview family members and healthcare professionals as much as possible during the same week as the patient participants.

The first author interviewed all participants, which provided a relative constant factor in the research process and was important for building trustful relationships with participants (Calman et al. 2013). He wrote memos during all stages of the research process, with information about preliminary results, interview setting, and factors that (had) affected the interview process.

The semi-structured interviews lasted an average of 1 hour and took place between December 2010 and November 2012. Patient and family interviews took place at their living place, like home, hospice or home care institution, and they were able to speak freely without being disturbed. Most interviews with healthcare professionals took place at their work place and they were sometimes disturbed by telephone. There were no indications that these disturbances affected the interview. The main reasons for non-participation or drop out of healthcare professionals was a lack of time.

### Research ethics

According to Dutch law our study did not need an ethics review (CCMO), which was confirmed by the ethics committee of the Academic Medical Center, University of Amsterdam. Although severely ill patients may benefit from participating in research, risks include the potential for emotional distress in participants and the main researcher, and difficulties around serial consent (Calman et al. 2013; Fairhall et al. 2012). These risks were discussed within the research team and the main researcher was an experienced non-denominational chaplain in psychiatry. As a consequence, he was able to attune to the needs of patients and patients’ family members during interviews and telephone conversations. In addition, patients were informed about the possibility of approaching the project leaders when the interview would disturb them, and a precautionary measure was that, in case they felt disturbed, professional support would be offered to patients and family members.

Eligible participants received information about the study both by letter and orally, and when they took part in the study, patient and family participants signed and informed consent form. In The Netherlands healthcare professionals only have to give oral consent (CCMO). In a few cases patient participants were unable to sign an informed consent. In these cases the informed consent was read aloud and patients gave oral consent, which was audiotaped, and a witness confirmed that patients participated voluntarily. Issues of confidentiality were explained to participants and patient participants were asked for written and oral consent for interviewing their healthcare professional and/or their family members about them. In order to guarantee the anonymity of all participants in this paper, demographics are presented collectively (Table 1) . The characteristics of two participants in this paper were changed because otherwise, they may be identifiable for their healthcare professional if (s)he would read this paper. However, these changes did not affect the presentation of the findings in this paper.

**Table 1:** Characteristics of participants (n = 100)

	Patients (n = 29)			Family/friends (n = 19)			Professionals (n = 52)		
	Mean	SD	Range	Mean	SD	Range	Mean	SD	Range
Age	65.9	14.7	37–91	52.1	13.6	24–75	49.0	8.9	29–64

	Patients (n = 29)			Family/friends (n = 19)			Professionals (n = 52)		
	N	%		N	%		N	%	
Gender									
Female	14	48		12	63		36	69	
Male	15	52		7	37		16	31	
Spirituality									
Non	14	48		8	42		20	38	
Monotheistic^a^	7	24		7	37		18	35	
Spiritual/other	8	28		4	21		14	27	

^a^Christian, Jewish, Muslim

### Data analysis

The researchers thematically analyzed fragments, in which participants explicitly referred to the word “hope” and derivatives thereof, like hopelessness and despair, which include the word “hope” in the Dutch language. MAXqda software was used to analyze the data. The main researcher (E.O.) thematically analyzed all the interviews and six researchers (four females) with backgrounds in sociology/nursing, religious studies, general practice/ethics, humanistic studies/ethics, or neurology co-analyzed a total of twenty-six interviews. They thematically analyzed what participants said about hope and how hope played a role in their relationships with others. For examples of the coding process of the two main findings presented in this study, see Tables 2 and 3. The researchers discussed their analyses until they reached consensus.

**Table 2:** An example of the code tree: hope and power

Quotations	Themes	Hope associated with
“Well, that [church] was like: keep hoping, allow yourself time, be strong and powerful, things like that” (patient 21)	Keep hoping: allow time, be strong, powerful	Power
“She was so powerful because of her hope that she didn’t leave any space for saying goodbyes”. (nurse 15)	Powerful because of hope, no space for goodbyes	Power
Let’s say: that hope, which people have had, I also think it is a sort of motivator, a sort of power, like: I will go on”. (physician 10)	Hope is motivator, power	Power
“Hope is: I wanna stay alive. That will to survive is very strong in human beings, no matter how ill they are. They cling to that” (patient 6)	Hope: strong will to survive	Strong will -> power
“Hope also includes a desire and courage as well, you make an inventory of power, and hope also gives power” (physician 12)	Hope includes desire, courage and gives power	Power
“Some people [with hope] try to keep themselves on their feet with thoughts like: it’s not as bad as it seems. And: I want to do many things. I also think that we as healthcare professionals sometimes are more negative than patients” (chaplain 10)	Hope keeps people on their feet	Keeping yourself on your feet -> power?

**Table 3:** An example of the code tree: loss of hope and suffering

Quotations	Themes	Loss of hope/no hope associated with
Then they’ve lost hope (…) at the point that they say: ‘It’s ready now. I’m done with struggling’”. (nurse 6)	Lost hope: done with struggling	Struggling -> suffering?
[During desperate moments I think about:] losing control. That I won’t be any longer who I used to be. I think that’s the word. That I won’t have any control over my body. That you’re pissing, shitting and stinking” (patient 12)	Desperate moments: losing control over body	No control over body and losing yourself -> suffering
“First they hope for cure. Then you’ve lost the hope for cure and then you hope to live for another year (…). You may deny it to a certain degree but the disease’s nature is that that won’t work anymore at a certain moment” (physician 7)	Lose hope because of physical suffering	Suffering
About her hope, our puzzle is: how do you simultaneously acknowledge her suffering and take things over from her? (nurse 9)	Hope versus suffering	Suffering
“We [man with COPD and I] saw each other during coffee, tea (…). He had always played [a wind instrument] in orchestras (…) so that man misses that a lot [pause]. Well, he had fun at that moment [with me]. No hope, but now again he had hope” (patient 29)	No hope because he missed his favorite hobby --> hope again because he had fun	Missing favorite things, no fun -> suffering?

In order to respect participants’ subjectivity and acknowledge their interpretations of hope, which is important in narrative and phenomenological approaches, interpretations of participants’ hope and derivation of main themes were checked during telephone conversations or during subsequent interviews, for instance by asking questions such as: “Do I understand that correctly, or do I have to interpret that in another way?”

Transcripts were returned to participants and they were invited to add new information during telephone conversations or during next interviews. Memos were used to reflect, analyze and capture ideas on hope. Saturation was reached when no new themes appeared in the data. By that time, around twenty-four patient participants had been included. Inclusion of patients continued until twenty-nine patients had been included (Calman et al. 2013; Tong et al. 2007).

## Results

### Participants

Of the participating patients (n = 29), eleven suffered from incurable cancer, ten from severe COPD, and eight from severe HF. Nine partners, four friends, three children, two brothers, and one sister-in-law were interviewed (n = 19). Around one half of the friends/family members were interviewed together with the patient participants. The included healthcare professionals (n = 52) were twenty-four physicians, among whom general practitioners, geriatricians and specialist physicians, eighteen nurses, among whom community, hospice, and specialist nurses, and ten chaplains with a Protestant, Roman Catholic, non-denominational, humanistic, or Muslim background. See Table 1 for demographic information.

### Hope and suffering

When patients, patients’ family members and healthcare professionals in our study addressed hope, they often spoke about power. They also associated hope with suffering and loss of hope. For examples of the code trees, see Tables 2 and 3.

#### Hope, power and empowerment

Talking about hope, participants often referred to power and empowerment. For example, they spoke about the power and encouragement to cheer somebody up, or they addressed the empowerment of hope.

“It [*hope*] has to do with a sort of *power.*” (family 12)

“Everybody knows that life is temporary. But if you’re suffering heavily, you should not emphasize that side in relation to your patient, is my opinion. You should rather give your patient hope, offer him something to cheer him up by telling him: ‘Have confidence! Be open for the *forces and powers* that accompany you’.” (patient 6)

“I think it is very good to *empower* a patient’s hope, for instance when there is a realistic chance that their hope will come true.” (physician 6)

Healthcare professionals in our study described hope in relation to a powerful bonding between specialist physicians and patients. One of them told about a patient’s hope for an experimental treatment. He explained how the patient’s hope was nourished by the bonding with the patient’s oncologist. A participating nurse expressed the power of hope in physicians, which affected physicians’ communication with patients.

“His hope is that it will help. Well, that absolutely is false hope. Because he gets an experimental chemotherapy and in my view the effect is zero. (…) The attachment of this man to his oncologist is so *powerful*! You can’t come in-between. It’s such a *powerful* relationship because that man has been treated for such a long time by this oncologist.” (physician 4)

“It is of course very interesting how the hope of the healthcare professionals relates to the hope of the patient (…). When the physician is so hopeful and is *empowering* the patient, like ‘let’s go for it,’ it offers no space for me to discuss with the patient that things aren’t going well (…). I think that is very *powerful* in physicians, that they want to be the messengers of good news and cure. (…) So they first have to accept that their own hope is dashed and then they even have to tell it.” (nurse 15).

The most powerful hope, according to participants, was hope for cure, which was mainly present in hospitals. One physician told how patients sometimes entered his hospice, having left the powerful and active atmosphere of hospital. A patient participant expressed how power and hope related to the hospital setting.

“[*Entering hospice,*] you’re leaving that active, powerful atmosphere [*of the hospital*]. That’s a line of fracture, definitely. So then the big hope has evaporated, the big hope for cure” (physician 10).

“How do they [*healthcare professionals in hospitals*] offer hope? Well, how do you say that? They just have the *power*: with their big buildings, their white coats, their education, their research studies, their medicines” (patient 12).

One participating physician told how she searched, beyond hope for cure, for “second best” hopes, like hope to see the daffodils grow or to experience the birth of a grandchild:

“So these kinds of new little hopes arise when the *big hope for definitive cure*, when that door has been closed. Then, a circuit of *second*-*rate little hopes* arises” (physician 12).

#### Loss of hope and suffering

When discussing hope, many participants spoke about suffering and the loss of hope. One nurse told how she cared for a cancer patient, whose family kept hope for living longer. She and her colleagues, however, wanted to treat the suffering.

“But at a certain moment they [*family*] switched over, like: ‘He’s got such a shortness of breath, that’s *not comfortable* anymore. So he may have to use the morphine injections.’ So we immediately jumped at that, because we actually had wanted that earlier because we saw that he was about to *suffer* a lot. But well, because of *their hope*, like: ‘You never know!’ they were like: well, if we start with morphine, then it’s all done.” (nurse 3)

Another healthcare professional also addressed loss of hope and its relation to suffering. He described how a hopeless situation sometimes led to hope, when the emotional and physical suffering were treated well.

“And people start to feel better and *less burdened*. Better pain medication, better treatment of *underlying suffering*, et cetera. Well, you can’t stop the disease: that process will continue. But you may introduce a temporarily stable phase (…), which leads to *hope*, like: hey, it’s not as *hopeless* as it seemed.” (physician 13)

Healthcare professionals not only tried to relieve the suffering but also to take seriously the loss of hope and suffering. One of them, for instance, tried not to downplay her own emotions while facing the loss of hope, while another participant expressed that suffering and hopelessness could not be solved by one simple answer. A patient participant stated that his psychologist was open for his hopelessness, which helped him.

“You reckon with the fact that sorrow or emotions come up while losing hope, and I’ve learned to allow that, not to immediately downplay that” (nurse 11).

“To be able to deal with that suffering, that hopelessness of life (…) [*you should realize*] that there is never one appropriate answer” (chaplain 9)

“She is open for that [*hopelessness*], you know. She immediately takes that seriously” (patient 1).

### Hope and suffering within relationships

Participants had different experiences with hope and suffering. Whereas some participants emphasized suffering, others emphasized hope, and yet others tried to pay attention to both suffering and hope.

#### Patients—family members

Patients and family members sometimes emphasized different sides: either hope or suffering. One patient participant emphasized the hope to continue his life as long as possible, while his wife emphasized the suffering, which was not worth the hope:

Patient 17 (husband): “I *hope* that it [*my life*] will continue this way, as long as possible. Until I will fall down. (…). But she said: ‘Maybe you could have better died.’ But please not! This period is worth living it.”

Interviewer: “Okay. Well, how is that for you?”

Family 19 (wife): “Well, I thought: then he doesn’t have to dread it, then he won’t *suffer.* There are things going on constantly: that he feels sick, that he doesn’t feel well. And then I think: if that could have been spared him, I would have liked that better. But he says: this is better with respect to his experience. Well, that’s true.”

Another patient participant told how her parents kept hope and tried to encourage her, while she mainly experienced suffering and longed for recognition of her suffering:

Patient 21: “When somebody constantly tells you, like: you’ll make it, and you’ll reach it,’ that’s also what my mother constantly said. Like: ‘Oh, you’ll make it and you’ll get over it, and blablabla.’ But no! I won’t make it. My heart says: it’s over. I’ve fought so much. Every time again. Every time again. (…) I think that they [*parents*] *maintain hope* and want their daughter to continue her life. But well, that won’t happen. *That’s heavy*.

Interviewer: May I ask you something about that?

Patient 21: Yes.

Interviewer: What makes it heavy?

Patient 21: “Yes, well, what makes it heavy? [little pause] Every day with *pain*. The one day the pain is less than the other day. I have also fallen down here. Well, that was a big crash: the floor is as hard as rock. Well, the *pain that you’re suffering* in your heart, your kidneys.”

#### Patients and family members—healthcare professionals

Differences were also found between healthcare professionals one the one hand and patients and their family members on the other.

In one case the patient was interviewed together with her husband. She (patient 10) always hoped for a “better tomorrow”, whereas the cardiologist, according to the patient and her husband, was focused too much on the suffering. When the husband was interviewed alone during the first interview (because the wife was too ill), he stated that the cardiologist was not aware that they had already accepted the reality of suffering and dying:

Family 12: “[I told her:] ‘Believe me, we really know the facts very [emphasis added] well.’ That’s what we talked about during that conversation with her. I told her: ‘Please listen to us: everything has already been discussed.’ I told her: ‘Even the music [of the funeral] has been discussed. Is it clear how we deal with the situation?’ (…).

Interviewer: “Did she think that you had false hope? Was that your impression?”

Family 12: “Yes, indeed! That was what she’d been thinking. Well, [name wife] can talk in a very hopeful way: ‘What doesn’t work today, will work tomorrow.’ But sometimes she’s trying for a week to go outside. But she will go on! That’s what she’s trying. That is also hope: that you try to realize it. So the cardiologist was a bit like: o, well, you’re too hopeful about therapy and cure and so on. But no, [*we focus on*] what’s within our possibilities. She didn’t recognize that part we were living with.”

During the second interview they were interviewed together and the patient stated:

Patient 10: “Well, this is the life that has been given to me. The quality of life, well, yeah, that’s a bit less than in a healthy person. Well a bit less? Quite much less, I do know that. But well, it’s because of his help, that I’m able to do some things.”

They made clear to their cardiologist that she focused too much on the suffering and since then, the cardiologist took a different approach. “Since then, the relationship is very good” (family 12).

In another case a physician told about her hospice patient who hoped for a “miracle cure”. She tried to discuss with her patient what the patient “expected of her future.” However, this evoked anger because the patient felt approached only as sufferer:

“She said: ‘Everybody only tells me bad news. I don’t need to hear that because I will continue praying that a miracle will happen’. So then I told her: ‘I would like to talk about it at least once and after that I will stop nagging.’ So after that I haven’t talked about the future for a *very* long time.” (physician 3)

#### Reflections of healthcare professionals

Several healthcare professionals tried to pay attention both to hope and to suffering. One participating nurse spoke about one of her patients and tried to balance hope and suffering. Another healthcare professional asserted that hope and (the suffering of the) disease do not go along very well. He tried to pay attention to the symptoms but also tried to focus on hope.

“The degree you appeal to her power has to do with hope (…) but if you ask too much of her, then you would deny her suffering” (nurse 9).

“Disease and hope don’t go along very well. So it is the person and hope. It is the art of not focusing too much on the disease. You need to pay attention to that [*symptoms*], of course. But also take into account: what else is playing a role here?” (physician 15).

Some healthcare professionals expressed how they needed hope while facing suffering of patients. One of them stated:

“Nurses don’t find it easy to give chemotherapies because you make your patients ill and you decrease their quality of life, because you hope that they will have quality of life after their treatments” (nurse 13).

## Discussion

The findings suggest that palliative care patients, their family members and their healthcare professionals associate hope with power and empowerment on the one hand, and with suffering and the loss of hope on the other. The results also suggest that within relationships both hope and suffering may play a role and that healthcare professionals try to balance both sides.

### Moral theories

The empirical findings presented above will be further articulated from a moral point of view.

#### Hope: empowerment

The findings on hope, power and empowerment reflect social-psychological and critical-sociological perceptions of em*power*ment. Social psychological theories have described empowerment as a process of personal growth (Kuokkanen and Leino-Kilpi 2000), and self-efficacy of palliative care professionals may indeed increase their hope (Duggleby et al. 2009). From a critical-sociological theory perspective, empowerment means the liberation from ties of oppression and inequality (Freire 2004; Kuokkanen and Leino-Kilpi 2000), which is a useful concept in order to critically approach the oppression of dominant and powerful hopes, like the hope for cure. The critical-sociological theory perspective on empowerment may furthermore help to critically approach the powerful bonding of hope between specialist physicians and patients, which was also addressed by others (Buiting et al. 2011; The et al. 2000). Empowerment and hope in these cases entail deconstructing dominant or taken for granted hopes and reconstructing new hopes, like one of the physicians in our study did (see above).

#### Suffering: compassion

The findings of suffering and the loss of hope, and taking seriously the suffering of another person, are reflected in the ethical concept of com*passion*. Martha Nussbaum (2001) defined compassion as “a painful emotion occasioned by the awareness of another person’s undeserved misfortune” (p. 301). It includes three cognitive beliefs: (1) the suffering is serious, (2) the person does not deserve it, (3) similar possibilities of sufferer and compassionate person—the suffering could happen to both of them (p. 304–327). Compared to empathy, compassion is not morally neutral: a torturer may be empathetic but is not compassionate (p. 329, 333). However, a critical comment should also be made: “If reason is used to justify the validity of compassion then compassion becomes a slave to reason. Subsequently, compassion becomes a slave to an abstraction, an idea of the good life like Nussbaum’s eudaimonistic conception” (Austin et al. 2013, 26). This remark should remind healthcare professionals that their own eudaimonistic evaluations and those of their patients may be different, which requires exploration in clinical practice.

#### Empowerment and compassion: solicitude

The data suggested that hope and suffering are related themes and others have also addressed these themes (Coulehan 2012; Eliott 2013). Several healthcare professionals tried to balance both, which did not mean that they always ended up in the middle but rather expressed their capacity to weigh both sides. Empowerment and compassion can be balanced in solicitude.

Erik Erikson’s reference to solicitude helps us to understand how solicitude includes the care for others, from which hope, power and empowerment may arise. According to Erikson, hope is the most basic and earliest human power that is rooted within caring relationships (Erikson 1994, 109–157). Animals also care for their young, “only man, however, can and must extend his *solicitude* over the long, parallel and overlapping childhoods of numerous offspring united in households and communities” (p. 130; italics ours). He concludes: “Care is the *widening concern* from what has been generated by love, necessity, or accident; it overcomes the ambivalance adhering to irreversible obligation” (p. 131; italics ours). For Erikson, hope remains a central factor in developing ego identity and autonomy (Wulff 1997, 373–381), which relates to empowerment.

Solicitude also includes compassion. Paul Ricoeur (Ricoeur 1994, 180–194) wrote that solicitude resembles friendship and aims at the good life. Friendship is characterized by “a fragile balance in which giving and receiving are equal, hypothetically” (p. 188). Solicitude, however, is first dissymetrical: the suffering of the other evokes my “benevolent spontaneity” and compassion. Although compassion may lead to dissymetry, “this is perhaps the supreme test of solicitude, when unequal power finds compensation in an authentic reciprocity in exchange, which in the hour of agony, finds regure in the shared whisper of voices or the feeble embrace of clasped hands” (p. 191). According to Ricoeur the re-establishment of equality requires the recognition of the superiority of the other’s authority, and a shared recognition of fragility and morality (p. 192). “A self reminded of the vulnerability of the condition of mortality can receive from the friend’s weakness more than he or she can give in return by drawing from his or her own reserves of strength” (p. 191).

In summary, solicitude includes the power of hope and empowerment, which are supported by mutually dependent relationships. On the other hand solicitude includes compassion, which is the shared recognition of fragility and suffering, in which the difference between giving and receiving is transcended.

### Strengths and limitations

One of the strengths of this study is that it empirically examined hope from several perspectives: patients, family members, and healthcare professionals. Another strength is that—because of the study’s longitudinal character—interpretations of hope during previous interviews were checked during subsequent interviews. In addition, the fact that one interviewer interviewed all participants reinforced trust between interviewer and participants, which is significant in longitudinal qualitative approaches (Calman et al. 2013).

However, our study only examined the micro level of individual stories and future studies should scrutinize hope at social levels, like others have done in hospitals (Mattingly 2010), and hope at political levels, which includes issues such as justice and allocation of (financial) resources. In addition, future research is necessary in other healthcare settings than palliative care, in order to sharpen or revise the findings presented in this paper, which may reinforce the external validity of the results found in our study. In the last place, the concept of suffering should be empirically scrutinized in future research.

## Conclusion

In conclusion, this study reinforced our understanding of hope in healthcare, which offered input for a relational ethics of hope. A relational ethics of hope consists of solicitude, in which empowerment and compassion are balanced. In our view, a relational ethics of hope thus implies that healthcare professionals sensitively attune to patients’ and family members’ suffering and hope. They should take seriously the suffering of patients and patients’ family members, for instance during physical deterioration, or when a meaningful event will not be achieved (compassion). On the other hand they should focus on hope and, for example, on what still can be done before dying and they should recognize patients’ own power in spite of severe illness (empowerment).

Such a relational ethics of hope relates to ideas that have been developed by care ethicists, who have addressed issues of relationships and power (Lindemann et al. 2009) and compassion with and recognition of vulnerability of others (Tronto 2009, 125–155). Future studies could compare, contrast and synthesize the concepts empowerment, compassion and solicitude with the concepts developed by care ethicists. Ultimately, we share the view of ethics of care that a relational ethics (of hope) should lead to reciprocal caring relationships between palliative care patients, their family members and their healthcare professionals.

## Electronic supplementary material

Supplementary material 1 (DOC 47 kb)
