# Supplementary material for: Solicitude: balancing compassion and empowerment in a relational ethics of hope—an empirical-ethical study in palliative care
Source: Med Health Care Philos. 2015 May 6;19:11–20. doi: 10.1007/s11019-015-9642-9 (PMC4805712; doi:10.1007/s11019-015-9642-9)
Supplement: Supplementary file 1 — Supplementary material 1 (DOC 47 kb) [file 11019_2015_9642_MOESM1_ESM.doc]

**Supplementary File 1.**

**Interview Guides.**

**Interview Guide Patient and Family Participants**

1. Opening
   1. Could you tell me what your first ideas were when you heard about the topic of this research: hope?
   2. Answers (and stories) were further explored by explorative questions: ‘What do you mean by …?’ ‘Could you tell something more about…?’ et cetera.
2. Concept of hope
   1. What does hope mean for you and what is its relation to hopelessness or despair?
   2. Are there other themes related to hope?
3. Dynamics of hope
   1. Have there been changes in your hope? *Prompts:* If yes, in what way? Did your hope, for example, increase, decrease, or remain the same? Could you tell me more about that?
   2. Have there been changes in your hopelessness? *Prompts:* If yes, in what way?
   3. Have there been changes in your despair? *Prompts:* If yes, in what way?
   4. Did other themes play a role in these changes? *Prompts:* If yes, which themes?
4. Social dimensions of hope
   1. What is the role of other persons in your hope? *Prompts:* which actions, responses or relationships help you to keep hope? Who threatens your hope? How did other respond to (changes in) your hope?
   2. Is hope the same for you, your family and healthcare professionals? If not, in what way is it different and how do you deal with that difference? If yes, how do you notice that it is the same?
5. Ethics of hope
   1. What do you see as a good way of dealing with (changes in) hope?
   2. What do you see as a good way of dealing with hopelessness?
   3. What do you see as a good way of dealing with despair?

*Prompts:* Could you give an example? Could you tell me more about that?

**Interview Guide Healthcare Professional Participants**

1. Opening
   1. 'What were your first ideas when you heard about the topic of this study, which is: hope in the lives of people who suffer from a disease from which they will sooner or later die?’
   2. Answers will be further explored by explorative questions: ‘What do you mean by …?’ ‘Could you tell something more about…?’ et cetera.
2. Concept of hope
   1. What is hope and what is its relation to hopelessness or despair?
   2. (How) does hope relate to emotions like fear, joy, sorrow, etc
   3. (How) does hope relate to religiosity or spirituality?
3. Dynamics of hope
   1. Do you see changes in hope in palliative care patients and their friends/families? If yes, in what way? *Prompts:* in what way does hope change? What are factors that influence changes in hope?
   2. Do you see changes in hopelessness in palliative care patients and their families/friends? If yes, in what way? *Prompts:* in what way does hopelessness change? What are factors that influence change in hopelessness?
   3. Do you see changes in despair in palliative care patients and their friends/families? If yes, in what way? *Prompts:* in what way does despair change? What are factors that influence changes in despair?
4. Social dimensions of hope
   1. Could you give an example of a situation in which hope of patients and family members was different? How did you deal with that difference?
   2. Could you give an example of a situation in which hope of palliative care patients and/or their friends/families was different from your own hope? How did you deal with that difference?
5. Ethics of hope
   1. What is a good way of dealing with hope?
   2. What is a good way of dealing with hopelessness?
   3. What is a good way of dealing with despair?

*Prompts:* Could you give an example? Which values are important for you in this example?

1. Hope and disease
   1. Do you see similarities and/or differences between (changes in) hope among different palliative care patient groups? (e.g. between severe COPD/other chronic diseases and cancer) *Prompt:* If yes, in what way?
2. Hope of professionals
   1. Could you give an example of things you hope for yourself? *Prompt:* think about hope in your own life and/or in your contact with palliative care patients or patients’ families or friends.
   2. What are situations in which you experience hopelessness or despair in relation to palliative care patients? *Prompt:* could you tell something more about that?
